# Update: Silicosis Mortality — United States, 1999–2013

**Published:** 2015-06-19

**Authors:** Jacek M. Mazurek, Patricia L. Schleiff, John M. Wood, Scott A. Hendricks, Ainsley Weston

**Affiliations:** 1 Division of Respiratory Disease Studies, National Institute for Occupational Safety and Health, CDC; 2 Division of Safety Research, National Institute for Occupational Safety and Health, CDC

Silicosis is a potentially fatal but preventable occupational lung disease caused by inhaling respirable crystalline silica (silica) (1). Chronic silicosis, the most common form, occurs after exposure to relatively low silica concentrations for >10 years. Accelerated silicosis occurs after 5–10 years of exposure to higher silica levels, and acute silicosis can occur after only weeks or months of exposure to extremely high silica concentrations (1). New national mortality data for silicosis have become available since a previous report on silicosis surveillance was published earlier this year (2). CDC reviewed multiple cause-of-death mortality files from the National Center for Health Statistics to analyze deaths from silicosis (*International Classification of Diseases, 10th Revision* diagnosis code J62: a pneumoconiosis due to dust containing silica) reported during 1999–2013. Each record lists one underlying cause of death (the disease or injury that initiated the chain of events that led directly and inevitably to death), and up to 20 contributing causes of death (other significant conditions contributing to death but not resulting in underlying cause). Available death certificates from 35 states were reviewed for the period 2004–2006 to identify occupations associated with silicosis among decedents aged 15–44 years. Results indicate that despite substantial progress in eliminating silicosis, silicosis deaths continue to occur. Of particular concern are silicosis deaths in young adults (aged 15–44 years). These young deaths likely reflect higher exposures than those causing chronic silicosis mortality in older persons, some of sufficient magnitude to cause severe disease and death after relatively short periods of exposure. A total of 12 such deaths occurred during 2011–2013, with nine that had silicosis listed as the underlying cause of death.

During 1999–2013, a total of 2,065 decedents had silicosis listed as the underlying or as a contributing cause of death (1,122 [54.3%] decedents had silicosis listed as the underlying cause of death) (Table). The annual number of silicosis deaths declined 40% from 185 in 1999 to 111 in 2013 (p-value for trend <0.001), but the decline appears to have leveled off during 2010–2013. The lowest number of silicosis deaths (88) occurred in 2011. Higher numbers of deaths occurred in 2012 (103) and 2013 (111), but remained within the 95% confidence interval predicted by the first-order autoregressive linear regression model used to evaluate trends for 1999–2013. Among all silicosis deaths, 47 (2.3%) decedents were aged 15–44 years; of these, 34 (72.3%) had silicosis coded as the underlying cause of death (Table). The annual number of silicosis deaths in persons aged 15–44 years varied and was 4, 0, and 8 in 2011, 2012, and 2013, respectively.

Death certificate review identified 62 silicosis deaths, accounting for 13.7% of the 451 reported silicosis deaths during 2004–2006. Of 39 (62.9%) decedents with silicosis listed as the underlying cause of death, three were aged 15–44 years. Entries on death certificates of these young decedents related to industry and occupation were classified* as miscellaneous nonmetallic mineral product manufacturing (stationary engineers and boiler operators), construction (brickmasons and blockmasons), and cut stone and stone product manufacturing (crushing, grinding, and polishing machine setters, operators, and tenders). These industries and occupations are well-known for their association with exposure to crystalline silica (1).

Silicosis mortality in the United States has declined over time (2,3). The continuing occurrence of silicosis deaths in young adults and reports of new occupations and tasks that place workers at risk for silicosis, including fabricators and installers of quartz-containing engineered stone products and workers employed to extract natural gas by hydraulic fracturing (4–7), underscore the need for strengthening efforts to limit workplace exposure to crystalline silica. Effective silicosis prevention strategies for employers are available from the Occupational Safety and Health Administration† and CDC’s National Institute for Occupational Safety and Health.§ State health departments can strengthen silicosis prevention efforts by identifying silicosis cases through review of state morbidity and mortality data and by investigating the circumstances surrounding silicosis cases.

## Figures and Tables

**TABLE:** Number of silicosis deaths, by age group, other selected characteristics, and year — United States, 1999–2013

	Age group					
	
	15–44 yrs		≥45 yrs		Overall	
			
Characteristic	Deaths	Underlying cause	Deaths	Underlying cause	Deaths	Underlying cause
**Total**	**47**	**34**	**2,018**	**1,088**	**2,065**	**1,122**
**Sex**						
Male	39	30	1,933	1,031	1,972	1,061
Female	8	4	85	57	93	61
**Race**						
White	37	27	1,727	927	1,764	954
Black	8	6	265	142	273	148
Other	2	1	26	19	28	20
**Ethnicity**						
Hispanic	9	7	131	83	140	90
Non-Hispanic	38	27	1,883	1,002	1,921	1,029
Unknown	0	0	4	3	4	3
**Year**						
1999	3	2	182	100	185	102
2000	5	5	146	66	151	71
2001	1	1	162	81	163	82
2002	5	4	141	85	146	89
2003	6	5	171	97	177	102
2004	2	0	163	76	165	76
2005	2	1	158	73	160	74
2006	6	3	120	64	126	67
2007	1	1	121	71	122	72
2008	2	2	144	83	146	85
2009	1	1	120	65	121	66
2010	1	0	100	52	101	52
2011	4	3	84	53	88	56
2012	0	0	103	58	103	58
2013	8	6	103	64	111	70
**P-value** *	0.45	0.33	<0.001	0.003	<0.001	0.004

* For trend during 1999–2013. Trends examined using a first-order autoregressive linear regression model.

## Footnotes

* The National Institute for Occupational Safety and Health Industry and Occupation Computerized Coding System was used to classify and code the industry and occupation according to the U.S. Census Bureau, North American Industry Classification System, and the U.S. Bureau of Labor Statistics, Standard Occupational Classification System, respectively. Additional information available at http://wwwn.cdc.gov/niosh-nioccs.

† Additional information available at https://www.osha.gov/pls/oshaweb/owadisp.show_document?p_table=standards&p_id=12716.

§ Additional information available at http://www.cdc.gov/niosh/topics/silica.
